# Using Human-Centered Design to Explore Potential Users' and Men's Views of New Injectable Contraceptives in Kampala and Lagos

**DOI:** 10.9745/GHSP-D-23-00215

**Published:** 2023-12-22

**Authors:** Holly M. Burke, Rebecca L. Callahan, Anna Lawton, Abigail Turinayo, Oluwatoyin Oyekenu, Sheila Niyonsaba, Oladunni Taiwo, Victor Muwonge Semaganda, Andy Awiti, Audrey Fratus, Fredrick Mubiru, Funmilola M. OlaOlorun

**Affiliations:** a Reproductive, Maternal, Newborn, Child Health, FHI 360, Durham, NC, USA.; b Product Development and Introduction, FHI 360, Durham, NC, USA.; c Design without Borders, Kampala, Uganda.; d SCOPE, Nigeria.; e SCOPE, Kenya.; f FHI 360, Kampala, Uganda.; g College of Medicine, University of Ibadan, Ibadan, Nigeria.

## Abstract

**Background:**

Injectable contraceptives are the most used method in sub-Saharan Africa. We conducted market research to assess potential user attitudes toward 4- and 6-month injectables. We also present user suggestions for marketing these new injectables once they are available.

**Methods:**

We implemented a 2-phase market research study from October through December 2021 in Kampala, Uganda, and Lagos, Nigeria. We conducted 11 focus group discussions (FGDs) with 51 participants in Kampala and 12 FGDs with 67 participants in Lagos. FGDs included current and potential injectable users and men stratified by marital status and age. Next, 23 women in Kampala and 24 in Lagos participated in cocreation workshops using human-centered design methods to explore marketing and communications strategies for each injectable. Data collection teams completed semistructured data extraction tables that were then analyzed thematically.

**Results:**

Participants liked both injectable options due to the reduced number of facility visits that would save time and money and increase privacy. Primary concerns included side effects, delayed return to fertility, cost, self-efficacy to self-inject, and stock-outs. Participants in Kampala preferred a shorter reinjection window (or “grace period”) because it is easier to remember and they assumed it meant a quicker return to fertility, but participants in Lagos preferred a longer window because it provides extra time for reinjection. Citing norms around women needing to get pregnant quickly after marriage, participants in both sites felt that the 4-month injectable would benefit young people with busy lifestyles or limited access to facilities, whereas the 6-month injectable would benefit women who already had children.

**Conclusions:**

We found that participants in Kampala and Lagos would prefer additional injectable options to meet the wide-ranging needs of users in different stages of their reproductive lives. Family planning program planners can apply the marketing insights we identified when these new injectables become available.

## Abstract (precis)

Although residents of Lagos and Kampala are interested in injectable contraception that lasts either 4 or 6 months, they have concerns as well.

## Teaser

**Key Findings:**

- Market research was conducted to assess users' attitudes in Kampala and Lagos about potential 4- and 6-month injectable products and found that current and potential injectable users and men liked both injectable products because of the reduced number of facility visits.
- Participants cited the option to self-inject as another benefit of the 4-month injectable but had concerns about side effects, cost, fear of improper administration with self-injection, and availability of the product because of continuous stock-outs of the 3-month injectable.
- For the 6-month injectable, participants reported the benefits of provider administration and longer length of action, which they also cited as a concern because of delayed return to fertility for those wanting to have children. Side effects and cost were also concerns.
- Participants felt the 4-month injectable would benefit young, busy people, whereas the 6-month would benefit women with children wanting to space births.

**Key Implications:**

- The new 4- and 6-month injectables explored in this study were appealing to women and men in Kampala and Lagos. Family planning program planners can apply the marketing insights we identified when these new injectables become available.

See related article by Callahan et al.

## BACKGROUND

Injectables are the most used contraceptive in sub-Saharan Africa, where they account for nearly 10% of all method use (inclusive of traditional methods) and more than a third of the modern method mix.^1^ The appeal of injectable contraception includes its discreetness, high effectiveness, and short-term action without requiring daily compliance and that it can be discontinued by users themselves.^2^^–^^4^

Despite being broadly adopted, discontinuation of injectable contraception remains high, with more than 40% discontinuing within 1 year across 19 countries.^5^ Users cite changes in menstruation and other side effects, concerns regarding return to fertility, and barriers to access as key motivations to switch methods or cease contraceptive use entirely.^2^^,^^6^^,^^7^

Depo-Provera (150 mg depot medroxyprogesterone acetate [DMPA] in 1 mL suspension) and its generic equivalents—all injected intramuscularly (DMPA-IM)—have long been the most widely used injectable products. However, Pfizer's recent introduction of a formulation of DMPA delivered subcutaneously (DMPA-SC) in a prefilled, single-use Uniject injection system—sold commercially as Sayana Press—aims to reduce some of the barriers to method continuation.^8^ DMPA-SC can be injected by users themselves, and studies from around the world have found this practice to be safe, effective, and appealing.^9^^–^^13^

Although DMPA-SC is labeled for a 3-month reinjection interval, a growing body of evidence suggests that a single, standard 104 mg dose may consistently prevent ovulation for at least an additional 4 weeks.^14^^,^^15^ A 750-participant study evaluating the effectiveness, pharmacokinetic profile, safety, and acceptability of Sayana Press when injected every 4 months for a total of 12 months resulted in zero pregnancies, and 90% of the participants reported being satisfied with the method.^16^^,^^17^ An extension of the reinjection interval would lower medroxyprogesterone acetate accumulation, possibly hasten return to ovulation and fertility, and reduce product and opportunity costs.^6^^,^^14^^,^^18^

Ongoing research is examining the potential for a 6-month injectable product. One of the most advanced leads, the repurposing of the existing DMPA-IM product as a subcutaneous injection, entered large-scale clinical effectiveness testing in 2022 [ISRCTN #62695528]. Although this product would be a traditional vial and syringe presentation with injection by a health care worker, user preferences studies have consistently found interest in a 6-month injectable product even if it did not offer the potential for self-injection.^19^^,^^20^

This article presents findings from market research in Kampala, Uganda and Lagos, Nigeria that assess potential user attitudes toward potential new injectable products providing 4 and 6 months of pregnancy protection. We also present user suggestions for marketing these products if and when they become available. This study is part of a series of activities to develop a regulatory strategy and explore the market implications of introducing multiple injectable products. The study was guided by human-centered design (HCD) thinking, which has been increasingly used in public health research to develop products and services responsive to user needs rather than ones to which the user must adapt.^21^^,^^22^ Core concepts or principles of HCD include empathy with users, rapid prototyping to gain insights, and tolerance for ambiguity and failure. The HCD framework typically involves 3 main phases: inquiry, ideation, and implementation. HCD emphasizes the use of participatory methods, a focus on the emotional triggers of behavior, and rapid iterations of data collection before reaching a final design solution. This study involved market assessments as part of the initial inquiry phase, which were then used to inform marketing workshops conducted in the ideation phase. The scope of this activity stopped short of implementation in the product development life cycle of the new injectable products, but the strategic communications outputs are anticipated to inform introduction and demand creation in the future.

We present findings from market research in Kampala, Uganda, and Lagos, Nigeria to assess potential user attitudes toward potential new injectable products providing 4 and 6 months of pregnancy protection.

## METHODS

We conducted this 2-phased market research, including qualitative group discussions and cocreation workshops in the greater Kampala Metropolitan Area, Uganda, and Lagos, Nigeria.

### Study Setting

Injectables are widely used in Uganda. Results from the Performance Monitoring for Action (PMA) program indicate that 14% of all women of reproductive age were using injectables in 2021—8% using DMPA-IM and 6% DMPA-SC. In Nigeria, injectable use is lower but has been growing. According to 2021 PMA data from Lagos, the prevalence of injectable use was 3.4% among all women of reproductive age—3% using IM and <1% SC. Injectables make up 43% of the method mix in Uganda (25% IM and 18% SC) and 17% in Lagos, Nigeria (16% IM and 1% SC).^23^^,^^24^ Using national- and district-level data collected between 2020 and 2022, estimates indicate users chose self-injection as their method of administration at approximately 7% of DMPA-SC clinic visits in Nigeria and 14% of visits in Uganda.^25^ Given its potential advantages, each country has a DMPA-SC task force of national stakeholders focused on product scale-up. In addition, the selected sites are accessible urban centers that offer the opportunity for peri-urban and rural representation without the need for lengthy travel.

### Eligibility and Sampling

The first phase of the research involved focus group discussions (FGDs) with 3 populations in each country: current users of injectable contraception, potential users of injectable contraception, and men who had not undergone a vasectomy and were in a sexual relationship with a woman. Potential users of injectables could be users of a modern contraceptive method other than injectables or sterilization, previous injectable users willing to use again, or current nonusers of any method willing to consider DMPA-SC. For all groups, individuals were eligible for the study if they were at least age 18 years, willing and able to provide informed consent, and willing to be audio-recorded (Table 1).

**TABLE 1.** Eligibility Criteria by Study Population for Market Research on 4- and 6-Month Injectable Contraceptive Methods, Kampala, Uganda, and Lagos, Nigeria

Study Population	Eligibility Criteria^a^
Phase 1: Focus group discussions	
Injectable users 18–24 years	Current users of DMPA injectables
Injectable users 25+ years	
Potential injectable users 18–24 years	Current users of modern methods other than injectables or sterilization Previous injectable users willing to use again Non- or never-users willing to consider DMPA injectables
Potential injectable users 25+ years	
Men 18–24 years	Men who have not undergone a vasectomy and were in a sexual relationship with a woman
Men 25+ years	
Phase 2: Cocreation workshop	
Recruited from focus group discussions	Injectable users Potential injectable users
Newly recruited from health facilities	

Abbreviations: DMPA, depot medroxyprogesterone acetate.

a In addition to the criteria in the table, all participants needed to be at least aged 18 years, provide informed consent, and agree to be audio-recorded.

We purposively recruited samples of women and men in each country stratified by marital status, age (18–24 years and 25 years and older), and for women, whether they were currently using an injectable. Using recruitment scripts, clinic staff informed women of the study and directed those interested to the study team. In Kampala, women were recruited with the help of staff at 2 health facilities in the Greater Kampala Metropolitan area—1 urban, high-volume facility and 1 peri-urban facility with lower volume. Men were recruited from community sites, including a public transport station. In Lagos, women were recruited with the help of staff at 5 health facilities in the peri-urban Agege area. Men were recruited with the help of the community mobilizer and 3 social mobilizers from the local government area who worked with the different community groups (e.g., transport associations, trade groups including mechanics, apprentices, and community leaders) to select men stratified by age and marital status. Women and men were recruited from different locations (facilities versus community sites) because, according to the clinic managers at facilities in both study sites, women often obtain injectables at health facilities without being accompanied by their partner. Therefore, men would be more efficiently recruited in community sites. Based on evidence that 80% of saturation in qualitative themes can be achieved with 3 to 6 FGDs,^26^^,^^27^ we aimed to conduct 4 FGDs with each subpopulation across both countries.

The second phase of the research included a 1-day cocreation workshop in each country. The workshops were intended to complement the FGDs with a focus on potential communication and marketing strategies for the 4- and 6-month injectable products. Workshop participants were women at least aged 18 years, both with and without prior injectable use experience, and open to using DMPA-SC. The target sample size for the workshops was 24 women selected purposively, including half who had participated in the FGDs and the other half newly recruited via the methods described earlier.

### Data Collection

The FGDs were conducted between October and November 2021 by trained staff from the design firm study partners, Design without Borders in Uganda and SCOPE in Nigeria. The Kampala FGDs were facilitated in person, whereas the Lagos FGDs were facilitated through video conferencing due to COVID-19 travel restrictions. All FGDs were conducted in either English or the local language, Luganda in Kampala and Yoruba in Lagos, depending on participant preference. The FGDs followed a semistructured discussion guide (Supplement) that explored drivers and barriers of uptake for existing contraceptive methods; attitudes toward and preferences for a new, generic 4-month self-injectable DMPA-SC; and views on a potential provider-administered 6-month injectable. Lines of inquiry focused on preferred method characteristics such as provider versus self-injection, reinjection window or the grace period for repeated injections, potential side effects, and time to return to fertility. Participants were shown images of calendars (Supplement) to illustrate the duration for the various injectable options (3-, 4-, and 6-month injectables) that would be discussed, as well as the reinjection windows (or “grace period”) for the 3- and 4-month injectables. In keeping with the principles of HCD, the discussions included several participatory activities, including journey maps to explore motivations, information needs, access considerations, and other concerns with method use; emotion cards to facilitate reactions to product concepts; and scenario probes to help assess drivers of contraceptive decision-making. Four personas were used to explore participants' perspectives on what kind of women would be best suited for the new injectables. For example, “Vivian” is a 20-year-old married housewife with 2 children who has never used a modern contraceptive method, and “Sharon” is a 32-year-old employed married woman with 4 children who currently uses DMPA-SC. The FGDs were conducted in private rooms in the health facilities in each country. The discussions were audio-recorded, and the data collection teams took detailed notes.

Phase 2 cocreation workshops were held in person in December 2021 and conducted in a mixture of Luganda and English in Kampala and Yoruba and English in Lagos. The design firm facilitators used a variety of participatory techniques to explore the foundations for a marketing and communications strategy for the 4- and 6-month injectable product concepts. Using data from the FGDs, the facilitators used user journey mapping, idea generation, mood boards, and storytelling to engage workshop participants to validate the target audiences for the new injectables, refine the product value propositions, and determine the most appropriate communication styles and channels to reach potential users.

All participants in both the FGDs and ideation workshops gave their written consent, received compensation for their time and travel, and were provided refreshments. Participants in the ideation workshop also received lunch.

### Data Analysis

Data from the FGDs were analyzed and interpreted on a rolling basis by the design firm teams in each country. The teams completed semistructured data extraction tables that followed the structure of the discussion guides with sections to record verbatim comments from participants and segmented by theme. The tables were completed in English using detailed notes collected during the FGDs, supplemented by the audio recordings. Data from the cocreation workshops were similarly captured through detailed notes and photos, on flip charts, and through participant-completed notes. The design firms developed reports summarizing the outputs and findings of both research phases.

An analyst (AL) extracted the findings from each of the Phase 1 reports and created thematic data tables in Miro (visual collaboration software) to organize and compare findings across the 2 research sites. We stratified participants by age and marital status to ensure that all groups were included in the study, but we did not analyze the data by these participant characteristics. The organization of themes for Phase 1 findings formed the scaffolding for the analysis of Phase 2 data. Phase 2 data were extracted from the reports and added to existing themes along with illustrative quotes when available to either support or contrast earlier findings. The other co-authors reviewed the data tables and provided inputs into the themes based on their experience collecting the data or reading the reports.

### Ethical Approval

Ethical approval for this research was granted by the AIDS Support Organization Research Ethics Committee and Uganda National Council for Science and Technology in Uganda, the Lagos State University Teaching Hospital Health Research Ethics Committee in Nigeria, and FHI 360's Protection of Human Subjects Committee in the United States.

## RESULTS

We conducted 11 FGDs with 51 participants in Kampala and 12 FGDs with 67 participants in Lagos (Table 2). Twenty-three women participated in the ideation workshop in Kampala and 24 in Lagos.

**TABLE 2.** Number of FGDs and Participants for Market Research of 4- and 6-Month Injectable Contraceptive Methods

	Kampala		Lagos	
	FGDs	Participants	FGDs	Participants
Injectable users 18–24 years	2	10	2	9
Injectable users 25+ years	2	10	2	10
Potential injectable users 18–24 years	2	10	2	12
Potential injectable users 25+ years	2	10	2	12
Men 18–24 years^a^	1	3	2	12
Men 25+ years^a^	2	8	2	12
Total	11	51	12	67

Abbreviation: FGD, focus group discussion.

a Male FGDs in Lagos were stratified by marital status. In Kampala, all participants in the 18–24-year-old male FGD were unmarried; one of the male 25+ groups included all married participants and the other included some unmarried and some married men.

### Perceived Benefits of a 4-Month Injectable

Both women and men in Kampala and Lagos preferred the 4-month DMPA-SC over the 3-month DMPA-IM because the former would require fewer facility visits and offered the option to self-inject (Table 3). The shorter reinjection window (or grace period) of the 4-month was perceived as another benefit among Kampala participants but not by those in Lagos.

**TABLE 3.** Preferences for 4-Month and 6-Month Injectable Contraceptive Method

	Both	Kampala	Lagos
Perceived benefits of 4-month injectable	Fewer facility visits Offered option to self-inject	Shorter reinjection window (grace period)	–
Perceived benefits of 6-month injectable	Fewer facility visits Administered by health worker	Intermediate duration	Long duration
Concerns about 4-month injectable	Side effects Delayed return to fertility Cost Self-efficacy to self-inject Stock-outs	–	Shorter reinjection window (grace period)
Concerns about 6-month injectable	Delayed return to fertility Side effects	Cost	–
Target audience for 4-month injectable	Younger Busy lifestyles Limited access to facilities	Unmarried Without children Do not want long-acting reversible contraception Frequent sexual activity	Married and unmarried Wanting to space children
Target audience for 6-month injectable	Older Married Has children In committed relationship Frequent sexual activity	Taking other medications	Young, single mothers wanting to avoid frequent facility visits
Reasons for preferring 6-month injectable over 4-month injectable	Longer duration Fewer facility visits Easier to remember reinjection interval Provider administered Easier to differentiate	–	–
Reasons for preferring 4-month injectable over 6-month injectable	–	Intermediate duration	Broader appeal
Ideal duration of injectables	6–12 months	–	–
Ideal number of injectable options	As many as needed to meet needs of users	2 options (4- and 6-month) up to 12 options	3 options (3-, 4-, and 6-month)

#### Fewer Facility Visits

Across Kampala FGDs, participants perceived the 4-month injectable as more cost effective and time saving, given the fewer annual doses one would receive compared to the 3-month as described by a participant.

*In this struggling economy we have to plan and budget for whatever we can in advance now, anything that can help us save. So, planning for fewer visits would be an advantage!* —Kampala, married, injectable user, 18–24 years

For Kampala men, the key selling point was the reduced costs due to fewer facility visits. For those in committed relationships, this was especially valued due to their role in facilitating transportation costs to health facilities for their partners. Participants in Lagos also felt that fewer facility visits would make it easier to calculate or remember return dates for reinjection. Participants in both countries felt the 4-month injectable offered greater privacy due to fewer chances of being seen at a facility. Lagos participants noted that fewer visits would especially offer privacy for women whose partners and spouses were unaware of their family planning (FP) use.

#### Option to Self-Inject

Though not different from 3-month DMPA-SC, participants in both countries liked the flexibility of being able to self-inject or have a provider administer the 4-month injectable. Kampala participants felt the self-injection option offered privacy for those who experienced stigma in accessing contraception, such as adolescents and those unmarried. Lagos participants noted the 4-month was comparable to the 3-month DMPA-SC, oral contraceptive pills, and condoms in that it could be self-administered and required minimal engagement with health care workers if desired.

#### Shorter Reinjection Window (Grace Period)

In Kampala, the 4-month injectable's shorter grace period of 1 week was viewed as a unique selling point for the method compared to the 4-week grace period of the 3-month. Though the researchers did not present it this way, the shorter grace period was assumed to be an indication of quicker return to fertility. The 1-week grace period was also preferred because it was seen as simpler to understand and easier to remember.

In contrast, the shorter grace period was viewed as a point of concern among Lagos participants. Most Lagos respondents were in favor of an injectable with a longer grace period, such as 4 weeks, because it gave them the flexibility to manage issues around privacy, such as facility visits without their partners' knowledge and other competing priorities. Participants noted that a longer grace period would be preferred by people with busy schedules having to find the time within the window to receive their next injection.

*Given a choice, it is the one with a longer grace period that we will pick.* —Lagos, consensus by unmarried, injectable users, 25+ years

Only 2 of the Lagos groups (injectable users aged 18–24 years and 25+) expressed indifference to the grace period as they were used to going to the facility immediately when their method was about to expire.

### Concerns About 4-Month Injectable

In both countries, the primary concerns about the 4-month injectable were side effects, delayed return to fertility, cost, and concerns related to potential self-administration, especially low self-efficacy to self-inject and safely store the product. Additionally, participants alluded to continuous stock-out of the 3-month product and wondered if the 4-month would be the same.

#### Side Effects

Participants from both countries were concerned about side effects of the 4-month injectable. In Kampala, participants wanted to know whether the 4-month would have the same side effects as the existing 3-month and how long these side effects would last.

*What are the advantages or disadvantages of the method? Does it cause frequent periods?* —Kampala, married man, 25+ years

Participants in Kampala described being able to withstand effects like prolonged bleeding, abdominal cramps, and loss of sexual desire for no more than 14 consecutive days. Further, some participants expressed concern about side effects increasing with longer use.

*Side effects get worse with regular reinjection—there is need to leave the body to rest! (for at least 3 months each year).* —Kampala, married, potential user, 18–24 years

In Lagos, when asked about the value of potentially reduced side effects with the 4-month injection, participants said that women would overwhelmingly choose the injectable with fewer side effects regardless of the duration. Indeed, uncertainty around side effects was cited as the top deterrent to using the 4-month injectable.

*The injectable with fewer side effects will be chosen even if it is 3-month injectable which allows for more hospital visits than the 4-month one. Thus, having fewer side effects supersedes the advantages of fewer hospital visits.* —Lagos, married, injectable user, 18–24 years

Lagos participants also wanted assurances that side effects would be mild or manageable, especially impact on menstrual bleeding and weight. According to participants, health care workers were their most trusted information source about contraception, so they should be well informed about the product to assuage any concerns potential users may have around side effects.

*In a situation like this I think it is better we meet someone that is more professional in this field to enlighten us.* —Lagos, unmarried, injectable user, 18–24 years

Several participants in 1 Lagos FGD said they would want to use the new method themselves first before recommending it to others, as they wanted to be sure the side effects were minimal and the cost was manageable.

#### Return to Fertility

For most Kampala participants, the optimal time to wait for fertility to return after discontinuing a method was between 7 and 14 days. Lagos participants felt information about side effects of the 4-month injection was most important, followed by information on return to fertility. Although they did not mention an optimal time, during discussions about a potentially quicker return to fertility for the 4-month compared to the 3-month, participants from 3 of the 4 Lagos FGDs said they would prefer the injectable with a longer return to fertility, especially when they did not want to get pregnant.

#### Costs

Participants in both countries were concerned about how much the 4-month would cost, with most desiring it to be free.

*It should be free … most girls cannot even afford sanitary pads …* —Kampala, unmarried, potential user, 25+ years

Kampala participants expected that, in public health facilities, the 4-month injection should be provided for free or with a small charge for provider administration as is currently done with the available 3-month option. Several Lagos participants said they were currently getting their method for free, and thus, their preference was to get the new injectable for free as well. Participants expected costs to be higher in private facilities due to their perceived profit-making agenda. According to Kampala participants, the 3-month injection was sold for an average of 5,000 Ugandan shillings (UGX) (∼US$1.50) in private clinics and pharmacies.

In both countries, some participants said they would be willing to pay for the 4-month because it would require fewer facility visits. Some Kampala participants also said they thought it logical that the 4-month would cost more than the 3-month and that they would be willing to pay a higher price for the former. Among Kampala participants willing to pay for the 4-month injectable, the suggested cost was not more than UGX2,000 (∼US$0.50) for rural areas and UGX7,000 (∼US$2.00) for urban areas. The highest cost suggested among participants in Kampala came from unmarried men aged 18–24 years who thought that UGX20,000–UGX25,000 (∼US$6.00–US$7.00) would be understandable for provision in private hospitals.

For Lagos participants who said they were willing to pay, the average price suggested was 500–1,000 Nigerian naira (∼U$1.20–US$2.40). Some male participants noted that because women were already paying for FP, cost was not a problem. Lagos participants said they were willing to pay if the method was guaranteed to be available.

#### Self-Administration

In both countries, most participants said they would opt for provider administration over self- injection because of fear of needles and incorrect administration.

*It is better for some women to go to the health center and collect the injection because some people are careless and they will abuse it (self-injecting).* —Lagos, unmarried, injectable user, 25+ years

In Lagos, some were concerned that improper administration would lead to infection and that women might forget to self-inject due to the “long” duration of effectiveness and then get pregnant. The absence of regular check-ups and interaction with health care workers with the longer intervals between facility visits was also noted as a concern.

Male respondents in both countries also preferred provider administration because they would rather have the liability for any mistakes to fall on the professionals at the facility. Some men also did not trust their partner to effectively self-inject.

*If I don't trust her to put on the power generator for fear of hurting herself, why would I entrust her with injecting herself?* —Lagos, married man, 25+ years

Difficulty finding a private place to store and administer the method themselves was also cited as a concern. Lagos participants wondered how “low-income” families who might have children and limited space would keep the injection safe.

*Self-injecting would be tricky especially for women in houses where there are children. How do you ensure that due to carelessness the needles are not left around.* —Lagos, married, injectable user, 25+ years

### Target Audience for 4-Month Injectable

In general, participants in both countries felt that the 4-month injectable would benefit “all kinds” of women from diverse backgrounds with varying needs. However, they felt that those who were young, seeking short-acting methods, with busy lifestyles, or with limited access to facilities would especially benefit. Some of the reasons participants thought the 4-month injectable would appeal to these groups differed by country.

In Kampala, participants felt the 4-month injectable would benefit young, unmarried women who did not have children and did not desire long-acting reversible contraception (LARC), which they perceived to negatively affect fertility. These women were thought to have independent decision-making power and high frequency of sexual activity. Participants thought the 4-month injectable would save these women money with fewer doses required. Participants also felt that youth would benefit from the privacy of self-injection because they could face stigma when accessing FP. The perceived quicker return to fertility of the 4-month injectable was also seen as a benefit for young women when they were ready to have children. Men also thought the method suitable for younger women, especially those pursuing education, potentially in an environment with more sexual encounters, or where an unplanned pregnancy would hinder their goals.

In contrast, participants in Lagos felt that women with active sex lives would prefer products with longer durations of effectiveness. They felt the fewer facility visits of the 4-month would appeal to busy women, both married and unmarried.

*It (4-month injectable) is suitable for married career women because it can be stored and readily available at home, so can be easily self-administered even though they are busy.* —Lagos, unmarried, potential user, 18–24 years

Like in Kampala, participants in Lagos felt that self-injection would benefit young women because it allowed them to avoid health workers who “looked down on them” for using FP. Lagos participants also felt that the 4-month would appeal to those who wanted to space their children because self-administration meant fewer visits to health facilities.

### Perceived Benefits of a 6-Month Injectable

Most participants in Kampala and Lagos were excited at the prospect of a 6-month injectable that would require fewer facility visits, save users time and money, and increase privacy compared to the currently available 3-month injectables. Another perceived benefit of the 6-month injectable was it was administered by a health worker, which they perceived to be safer.

#### Fewer Facility Visits

Kampala participants noted that it could be difficult to find time to go to the health facility, especially for employed users, so the fewer doses required of a 6-month product would be a benefit. Participants also felt the 6-month would offer privacy to users because of the fewer required trips to the facility and because partners could not detect it. Male participants felt that costs over time would be lower for the 6-month and that it would be easier to remember the reinjection date compared to available injectables.

Lagos participants felt that 2 facility visits per year would be a benefit of the 6-month because it would “allow time for other things,” such as academic aspirations, financial stability, family, and career and business progression, and was “long enough to allow you to plan your life.” As in Kampala, Lagos participants also thought the method would benefit women who wanted to use contraception without their partners knowing.

#### Length of Action

The 6-month injectable was generally perceived among Kampala participants as a suitable “in-between” option (not too long and not too short). Participants felt the intermediate duration would allow users to delay their next pregnancy without having to use LARCs. This was seen as an advantage because of participants' belief that prolonged use of long-acting methods caused adverse side effects, including a delayed return to fertility or sterility.

However, in Lagos, participants viewed the 6-month injectable as comparable to implants and intrauterine devices because of its long duration and reduced number of facility visits. These features were viewed positively as offering “peace of mind” because the long duration, in participants' opinion, reduced the chance of experiencing unplanned pregnancies. Lagos participants further noted that the 6-month duration would be long enough to allow for “planning of one's life,” support child spacing, and allow for regular intimacy.

#### Administered by Health Care Workers

Though some Lagos participants mentioned that lack of a self-administration option meant that it could be time consuming to access the method at health facilities, this sentiment was in the minority. Most participants in both countries said they would prefer provider-administered injectables because they doubted their ability to correctly self-administer. Lagos participants noted that administration at the health facility would “guarantee safety.”

### Concerns About 6-Month Injectable

Participants in both countries expressed concern about the return to fertility and wanted to know whether users of the 6-month injectable should expect a delayed return to fertility and whether side effects would last for the whole duration of use. Kampala participants were also concerned about the cost of the method.

#### Return to Fertility

Kampala participants were concerned about whether there would be an even further delay in users' return to fertility, given the longer duration of effectiveness of the 6-month injectable compared with existing injectables. Male participants in Kampala were especially concerned about the potential delay in fertility return and felt that a 6-month duration would be too long for a couple who were considering having their first child.

Lagos participants also felt that lack of reversibility of the 6-month injectable could be problematic for people who changed their minds and wanted to get pregnant before 6 months. Some noted that the method may not be suitable for young women who were uncertain about when they wanted to become pregnant.

#### Side Effects

Kampala participants wanted to know whether side effects of the 6-month injectable would be the same as the existing 3-month injectable and whether a long duration of side effects might affect one's productivity, create conflict between partners if they failed to conceive, and result in costs incurred with managing side effects.

*I already suffer bleeding for all the 3-months with the injectable I'm using! I worry this 6-month is even worse!* —Kampala workshop participant

Side effects of most concern were prolonged bleeding, delayed return to fertility, abdominal cramps, and loss of sexual desire. However, participants also felt that if side effects were short lived, then the 6-month would be a highly desirable product.

*If I could bear with the other side effects [of 3-month product], I can bear with those for the 6-month injectable as well.* —Kampala, unmarried, injectable user, 18–24 years

Most Lagos participants did not express concerns about side effects, but some wanted reassurance that any side effects would be similar or less than those associated with the existing 3-month option. Some felt that the extended duration meant they needed to think through and be clear about the implications before choosing the new method because they would “have to live with it for 6 months.” Some felt it would be best to consult with their partner as part of this process.

*I think some might actually think it might damage their body because of the long duration like the implant.* —Lagos, unmarried, injectable user, 25+ years

#### Costs

Kampala participants were concerned about the cost of the 6-month injectable, but they expressed a willingness to pay 50% more than the current price of the available 3-month injectable methods. In addition, they emphasized the need to distinguish the 6-month method as a twice-a-year product that had the added advantage of health provider administration with continued counseling and support throughout use. Participants felt this support would provide encouragement to the users and ensure their continued use of the method. Lagos participants were willing to pay the same price as the 3-month or slightly more for the 6-month injectable. However, they felt that it should be affordable for all women.

### Target Audience for 6-Month Injectable

Overall, participants in both countries thought the target audience for the 6-month injectable would include older, married women who already had children and were in committed relationships. Both felt it was not appropriate for young nulliparous women who were unsure of when they wanted to have children because of fertility concerns and the duration being perceived as too long. Kampala men and participants in Lagos also felt it was a good method for women with very active sex lives because of its long duration and reduced number of required clinic visits.

#### Older, Married Women With Children Who Want to Space Births

Participants in Kampala generally felt that the 6-month injectable was suitable for women aged 30 years or older who already had children because participants were concerned about potentially prolonged side effects. Given the potentially longer delay in return to fertility for the 6-month option, participants thought that these women would be able to effectively space their children without being exposed to the adverse side effects they associated with most LARCs.

Even though participants in both countries felt the fewer annual doses of the 6-month would enhance privacy among users, they thought that the method might also foster shared decision-making among couples. Kampala participants felt that women's use of the 6-month might involve their partners because of the joint decision-making surrounding the desired number and timing of children.

Lagos participants also felt the 6-month would be suitable for married women because the duration was “short enough” for those who wanted to get pregnant again. Participants thought the 6-month would foster shared decision-making through open conversations with one's sexual partner. During the workshop, participants evoked the term “ka jo lo,” which means “we should use it together,” during discussions about the 6-month method. Participants also felt that the 6-month could be advantageous for young single mothers who wanted to avoid frequent visits to the health facility.

#### Women With Active Sex Lives

Lagos participants thought the 6-month duration would appeal to women with “very active sex lives.” Kampala men felt the method was best suited to women who were not in committed relationships and who had unplanned sexual encounters because of the fewer facility visits.

*The 6-months method allows you to visit the hospital only twice a year, so this makes it a good method especially for those that are single.* —Kampala, unmarried man, 18–24 years

#### Women Using Other Medications

Kampala participants also saw the 6-month injectable as providing longer-acting pregnancy prevention relevant for women taking other medications.

*It will be beneficial for HIV patients because they would not have to worry about taking more medication often, in addition to their treatment*. —Kampala, married, potential user, 25+ years

### Ideal Duration and Number of Injectable Options

#### Preference Between 4-Month and 6-Month Injectables

In Kampala and Lagos, among both women and men, the 6-month injectable was preferred over the 4-month because of its longer duration, fewer required facility visits, easier-to-remember reinjection interval, and provider administration. Compared to the 4-month injectable, participants also felt that it was easier to differentiate the 6-month injectable from existing injectable durations.

*I will go for the one that would last for a long period of time.* —Lagos, unmarried, injectable user, 18–24 years

However, Kampala participants were concerned about women aged 25 years and older and who were not current users of injectables “leaping from 3- to 6- months” and said they would prefer an intermediate duration from the existing 3-month injectable to feel confident. Participants described the new 4-month injectable as the potential intermediate option that users could try to establish “compatibility with the body” before adopting a longer-acting method like the 6-month injectable. This sentiment was seen in participants' discussion about how the 6-month injectable would be “too much” for the persona “Vivian” because she had not previously used a modern FP method.

*It would be better for “Vivian” to first use a shorter method like the 3-month injectable so that she can see how her body reacts to it.* —Kampala, unmarried, potential user, 25+ years

The 6-month injectable was also received more positively than the 4-month by Lagos participants because it was deemed easier to differentiate from existing injectable durations. Participants remarked that it was difficult to differentiate between the 3-month and 4-month injectable products. Even those participants who mentioned liking the 4-month option wanted it to be introduced alongside the 6-month option. Only 1 group (injectable users aged 18–24 years) suggested a preference for the 4-month injectable due to the feeling that it would have broader appeal compared to the 6-month injectable, which was more suited to older women who already had children.

#### Ideal Duration of Injectables

Participants in both countries liked the concept of longer injectable methods (lasting 6–12 months), citing suitability for single mothers, education/career-oriented women, and married women with children due to reduced visits (meaning reduced costs and increased privacy), adequate child spacing, and ease of remembering reinjection.

Kampala participants viewed 6–12 months as a reasonable duration of pregnancy protection, with only a few preferring durations of 3–5 years. Participants felt that for married women aged 25 years and older who were using contraception to space children, shorter-acting injectables introduced greater risk of miscalculating or forgetting their reinjection date.

Contrary to biases about LARCs described earlier, Kampala participants noted that some younger unmarried women would desire a longer-acting method, such as the 6-month injectable, due to cost savings. Participants felt that because many younger women (defined by participants as aged younger than 25 years) were still in school or seeking work, they were not ready to have children, making the 6-month a desirable option.

Lagos participants believed that longer-lasting injectables would have interest across user groups, including career-oriented users, married women wanting to space births, and single mothers. The reduced frequency of facility visits was a key appealing factor as it could increase privacy and reduce costs.

*The 6 months is less stressful because it will be taken twice a year.* —Lagos, unmarried, injectable user, 25+ years

Two female participants in each age group (18–24 years and 25+ years) who were current self-injectors preferred the injection with the longest duration.

*It cannot stop me (6 months not being self-injectable), I will start using it if it comes out. Then anytime self-injection is available, I will then switch to doing it myself.* —Lagos, unmarried, injectable user, 25+ years

#### Ideal Number of Injectable Options

Participants in both countries suggested the more injectable options, the better to meet the needs of different users; most did not feel that confusion between products would be an issue.

*It can't confuse people because there's difference there [between the injectables] and explanation will be made.* —Lagos, married, injectable user, 25+ years

Kampala participants recommended that the number of injectable options should not be limited and that more options would meet different users' desires, needs, and goals, with some suggesting as many as 12 options. A few participants said that 2 options would be ideal to minimize potential confusion and that the 4- and 6-month injectables would be preferred over the 3-month injectable. The reason they suggested these 2 options was because they did not see much difference between the 3- and 4-month injectables, particularly regarding their duration of effectiveness. However, the consensus was that women of reproductive age would benefit from as many injectable product options as possible.

*It is impossible to determine an ideal duration because it varies for different people, their goals and needs…even an option of 1 week works.* —Kampala, unmarried, injectable user, 25+ years

Lagos participants were also in favor of having multiple injectables of different durations because it would offer women the freedom of choosing between the various options depending on their needs.

*Actually, it's people's choice, they will actually choose what they want and make their decision.* —Lagos, unmarried man, 25+ years

According to participants, the ideal scenario would be one where people could choose between a 3-, 4- and 6-month injectable options freely. The participants did not believe that it would be confusing to have multiple options.

*If you're not pleased with say the 4-month, you can go back to the 3-month, it's a matter of choice, it's not by force.* —Lagos, married, injectable user, 25+ years

### Marketing Messages for New 4- and 6-Month Injectables

Participants in both countries came up with similar marketing themes of autonomy/agency/power/control over unplanned pregnancies and, therefore, achieving one's goals, convenience, and flexibility because of choice of administration (self-injection or provider-administration) and the ability to change your mind or method (because it is a short-acting method).

Tables 4 and 5 summarize the marketing elements that were identified for the 4- and 6-month injectables, respectively. The positioning statement defines the main point about the product that the target audience needs to know, hear, and remember about the product. Brand pillars enable the product to stand out from the competition. For the target audience, these are the reasons to trust and choose the product. The brand promise is based on the understanding of the values, interests, strengths, and personal qualities of the target audience described. Finally, the brand personality includes a set of human traits and characteristics assigned to the product that should connect with the users' desired outcomes of using the new product.

**TABLE 4.** Marketing Elements for a 4-Month Injectable

Element	Kampala	Lagos
Positioning statement	The 4-month injection gives women the power and convenience to prevent unplanned pregnancy while they pursue their dreams.	The 4-month injection supports women in taking full charge of the prevention of unplanned pregnancies, by offering them flexibility and peace of mind in a package that they can choose to use safely and easily, anytime and anywhere.
Brand pillars	Method can be self-administered, which can ensure privacy particularly for those who face stigma accessing family planning. The shorter grace period of 1 week is perceived as indicating quicker return to fertility.	Control Method can be self-administered anytime, anywhere. Fewer visits to health facilities. Requires less administrative action in comparison to daily pills and shorter term injectables. Ease of use Easy to self-administer compared to other products in the market. Easy to store safely, use, and dispose. Reliability Guaranteed duration of effectiveness for 4 months.
Brand promise	The method will bring women no shame; it will foster excellence by giving women time and opportunity to grow and succeed at their plans, as well as giving them the flexibility to change their plans when they want to.	Contraception for the woman who wants full control.
Brand personality	The method needs to have an inclusive, aspirational, and creative personality. The tone of the communication of the method should convey ambition and offer a sense of confidence and ability to reach new heights. The style of the communication should portray real women from all parts of Uganda choosing the 4-month method in different seasons of life, such as university student at the start of the semester, home assistant starting a new job in the city, and a woman exploring a new relationship. Stay calm, there's no need to panic anymore. You hold the key - you decide. Focus on your dreams. Explore your options. Take your time, at your pace.	The following emotions should be associated with users of the method: Happy and calm because they are less worried when having sex. Hopeful due to availability of a solution to prevent unplanned pregnancies. Afforded more flexibility (reduced visits to facility) and additional flexibility for those who preferred self-administration. The tone should be: Friendly: relatable with a touch of humor. Uplifting: inspiring, affirmative, and supportive of women. Confident: open and proactively informative about the product.

**TABLE 5.** Marketing Elements for a 6-Month Injectable

Element	Kampala	Lagos
Positioning statement	Opportunity to create ideal child spacing at a reduced cost and with fewer visits to a facility.	A contraceptive that offers busy women the certainty they need to plan long term and the time to do what matters most.
Brand pillars	Low cost of being a twice/year method. Long-acting pregnancy protection without having to use a long-acting reversible contraception, which are perceived by some to have adverse side effects including a long return to fertility. No detectable presence in the body so can be used discreetly.	Liberating: only 2 facility visits per year and can be used discreetly. Certainty: long duration allows users to make long-term plans. Reliability: guaranteed duration of effectiveness for 6 months.
Brand promise	Will foster the healthy growth of one's family by providing a pregnancy prevention method for an adequate duration between pregnancies. This promise helps fulfill the parental desire of dedicated time to nurturing their children—more than providing basics but also building deep relationships with them.	Contraception for the woman who wants the certainty to pursue more in life.
Brand personality	This method needs to have a frugal, trustworthy, responsible, and family-oriented personality. The tone of the communication of the new 6-month method should therefore convey joy with a stress-free approach to family life, providing comfort and security to a home. The communication style should portray different stages of family life; from the couple deciding together, to the birth of the first child, and the steady growth of the family. Stress free and pocket-friendly. Providing a sense of priority and wise living. Dedication and strong commitment to a healthy family. We are stronger together. Open communication that is easy to understand.	The following emotions should be associated with users of the method: Peace of mind because of the long duration of effectiveness. Achieving the “good life” by supporting child spacing and reducing strain on resources. Safety because it is administered at a health facility. Allowing for regular intimacy due to longer duration. The tone should be: Friendly: relatable, with a touch of humor. Uplifting: inspiring, affirmative, and supportive of women. Assertive: reinforcing certainty and conviction of the woman's decision-making. Confident: open and proactively informative about the product.

## DISCUSSION

Using HCD principles, we conducted market research in Kampala and Lagos to assess potential user attitudes toward new 4- and 6-month injectable contraceptives and present user suggestions for marketing these new products once they are available. Participants liked both injectables because of the reduced number of required facility visits, which would save users time and money and increase privacy. The primary concerns about the injectables were side effects (most important for 4-month; second most important for 6-month), delayed return to fertility (most important for 6-month; second most important for 4-month), cost (4-month, both countries; 6-month, Kampala only), and concerns related to self-administration (4-month), and stock-outs (4-month). Interestingly, although participants identified self-injection as a benefit of the 4-month injectable, most said they would prefer provider administration due to perceived low self-efficacy to self-inject. This finding could be because of limited uptake of self-injection in the study settings and might change as the practice of self-injection becomes more prevalent and normalized.^25^ Other research has found fear of self-injecting to limit uptake of self-injection.^28^^–^^30^

We observed study site differences in preferences between a longer versus shorter reinjection window (grace period) for the 4-month injectable. A shorter reinjection window was perceived as a benefit among Kampala participants because they thought it would be easier to remember and because they perceived a shorter window to be an indication of quicker return to fertility after stopping the method. However, Lagos participants preferred a longer reinjection window because it would give people extra time and flexibility to get their next injection. In line with this finding, we observed site differences between participants' thoughts on the time it took for fertility to return after stopping injectables. Participants in Kampala valued rapid (less than 14 days) return to fertility after stopping injectables, whereas most Lagos participants perceived a longer return to fertility as a benefit. Data published after this present study showed that 3 versus 4 injections of DMPA-SC over 1 year resulted in a 1-month quicker return to ovulation. However, whether this 1-month difference will be meaningful for users is unknown.^31^

We also found a common belief among Kampala participants that LARCs were associated with a long return to fertility after method discontinuation and are generally associated with side effects that negatively affect fertility. However, implants and intrauterine devices have a much faster return to fertility (approximately 1–4 weeks after discontinuation), whereas DMPA injectables have one of the longest (approximately 9 months after discontinuation).^32^^–^^35^ Although this misperception was described during discussions of the 4- and 6-month injectables, it was most pronounced when Kampala participants described the 6-month injectable as a suitable “in-between” option that would allow users to delay their next pregnancy “without having to use LARCs.” This belief was not found among the Lagos participants, where the 6-month injectable was compared favorably to LARCs for its reduced number of facility visits. Marketing campaigns, as well as pre-service and in-service trainings for health care workers, should actively dispel misconceptions.

As is true for contraception generally, participants thought that more injectable options would be better to meet the wide-ranging needs of users in different stages of their reproductive lives.^36^^–^^38^ However, when asked about the 2 injectables being considered in this study, women and men in both settings preferred the 6-month injectable over the 4-month because of its longer duration, fewer required facility visits, easier-to-remember reinjection interval, and provider administration. Participants also felt the 6-month was easier to differentiate from the existing 3-month injectable options than a 4-month would be. In line with more options being better, some Kampala participants suggested the 4-month could fill the “leap from 3- to 6-month” for potential 6-month users, suggesting that people need to test the method first to ensure compatibility with their body.

Participants in both countries felt that the 4-month injectable would especially benefit young people with busy lifestyles or limited access to facilities. The self-injection option offered privacy and would allow users to avoid interacting with health care providers, which would appeal to some groups like adolescents who face stigma when accessing FP,^39^^,^^40^ but for other groups, would be perceived as a loss of support from a knowledgeable provider. Kampala participants felt that the 4-month injectable would appeal to unmarried women who did not have children, whereas Lagos participants felt that it could appeal to either married or unmarried women, with the defining characteristic being people who are busy. However, participants in both countries agreed that the 6-month injectable was for women who already had children and confirms norms around women needing to prove their fertility and get pregnant quickly after marriage or partnering.^20^^,^^41^^,^^42^ Participants also felt the decision to prevent pregnancy for 6 months should be made with the partner, thus suggesting that the 6-month injectable may be an opportunity to engage men in FP.

The marketing and communication insights gained during this research can be applied in FP programs in the study settings to reach potential users of these new injectables when they become available. Some of the insights were similar across the 2 settings, for example, describing the 4-month injectable as offering users flexibility and the focus of the 6-month injectable for child spacing. However, contextual nuances were apparent and could help program planners create tailored marketing campaigns specific to the study contexts. We do not know when these products will be available in the study sites; however, we believe that most of the insights generated through this research will remain useful even if the time is extensive because many of the insights were based on long-standing norms around FP (e.g., concerns about side effects and return to fertility). The exception was concerns around self-injection, as previously described.

### Strengths and Limitations

We conducted this study in 2 urban cities and purposively sampled participants from a few locations in each, thus limiting generalizability of findings beyond these settings. Further, 1 of the FGDs with men had a small sample size. Given the exploratory nature of this study, the discussion guides contained questions that were open-ended to facilitate discussion among the participants without trying to introduce preconceived ideas from the research team. However, this meant that some topics that were spontaneously raised in 1 group were not systematically addressed in the other groups. Further, the group discussion format may have introduced social desirability bias because people may want to please the moderators and/or other participants. We attempted to minimize this bias by using HCD approaches and experienced designers who were familiar with the study settings. The design teams used a variety of activities, including personas, that allowed participants to talk about other people and not their own behaviors or attitudes if preferred. The lack of verbatim transcripts limits the availability of quotations and our ability to quantitatively assess intercoder reliability; however, we checked reliability by discussing the data and arriving at consensus across our diverse research team.

Despite these limitations, our study has several strengths. Importantly, the use of HCD increases the participants' agency in problem-solving and challenges traditional ways of thinking, thus encouraging novel ideas to emerge and resulting in people-centered solutions that are more likely to be adopted.^43^ The study also benefited from implementation across 2 different settings, which allowed the results to be compared across settings to identify commonalities and differences.

## CONCLUSION

In conclusion, we found that participants in Kampala and Lagos would prefer additional injectable options to meet the wide-ranging needs of users in different stages of their reproductive lives. The new injectable products lasting 4 and 6 months explored in this study were appealing to participants. FP program planners can apply the marketing insights presented above when the new injectables become available.

## Supplementary Material

23-00215-Callahan-Supplement.pdf

## Notes

**Peer Reviewed**

**First published online:** December 6, 2023.
