# Supplementary material for: Using Human-Centered Design to Explore Potential Users' and Men's Views of New Injectable Contraceptives in Kampala and Lagos
Source: Glob Health Sci Pract. 2023 Dec 22;11(6):e2300215. doi: 10.9745/GHSP-D-23-00215 (PMC10749653; doi:10.9745/GHSP-D-23-00215)
Supplement: 23-00215-Callahan-Supplement.pdf [file 23-00215-Callahan-Supplement.pdf]

Supplement to: Burke HM, Callahan RL, Lawton A, et al. Using human-centered design to explore potential users' and men's views of new injectable contraceptives in Kampala and Lagos. *Glob Health Sci Pract*. 2023;11(6):e2300215. https://doi.org/10.9745/GHSP-D-23-00215

Guide for Focus Group Discussions with Contraceptive Users

Market Research on User Perspectives for a 4-month DMPA-SC Product

FGD Number: [__|__]

FGD Description: ________________________________

Date: ______________________________

Step 1: Obtain consent individually (separate document) and conduct demographic questionnaire individually (separate document)

Step 2: Convene group and conduct the discussion

[Begin recording here]

Introduction: Today is [date] and this is [Focus Group ID]. My name is ______________________. Thank you again for your participation in the discussion today. We are looking forward to hearing your thoughts on the questions we will ask you. As a reminder, your participation is completely your choice, your answers will be kept confidential, and you do not have to answer any questions you do not want to, or you may refuse to participate.

Please know there are no right or wrong answers or opinions about the topics we are discussing, so feel free to share your thoughts openly. During the focus group discussion, we would like you to share your thoughts and discuss this topic. My role as moderator will be to guide the discussion. I would ask that you do not share information discussed during this session with others outside the group. However, we cannot guarantee that information discussed in the group will not be shared, so consider this before discussing personal matters. As mentioned earlier, the discussion is going to be audio-recorded for documentation purposes. Because we are recording, it is helpful if you speak clearly, slowly and one at a time.

SECTION 1: Study Introduction

FGD Presentation // Interviewer: Use to guide all sections of the discussion

[Interviewer: Read the following information to the group. Pause for questions/confusion and attempt to clarify when possible. When introducing DMPA-SC/Sayana Press to the users, present a sample product or image of a product to aid in recall.]

I am here to talk to you today about injectable contraceptives, and in particular one that you may know of as [local name for DMPA-SC/Sayana Press]. It can be administered by a provider, or you can also self-inject. [Local name for DMPA-SC/Sayana Press] is currently labeled here in [Uganda/Nigeria] for preventing pregnancy for up to 3 months

SECTION 2: Getting to know the users

1. To start with, could you tell me a little bit about yourself?
    a. Where do you stay?
    b. Who lives with you there? [Probe for children, partner/spouse, etc.]
    c. Do you work outside the home? If so, what do you do?

SECTION 3: Understanding user perceptions around family planning

2. When I say "family planning" what is the first thing that comes to mind?
    a. What word(s) or terms do you typically use? [If no suggestions are made, probe around terms such as contraception, birth control, "the pill"]

Activity 1_ Image Association Cards // Interviewer: Lay out the image association cards (emotion cards) and ask users to pick one (or two) cards that represents how they feel about FP; ask each to explain their choice to the group.

    b. How do you feel about family planning? Why do you feel this way?

3. What family planning methods do you know/are you familiar with? [Note local names/phrases that may not be common, and probe as needed]

Activity 2_Showcase FP methods // Interviewer: Ask the users to list out all the FP methods they are aware of, irrespective of personal experience. As users name methods either a) present the physical method, b) present an image of the method, or c) list these out on card stock. Lay them out as identified for discussion and probe on those that are not mentioned.

4. You've mentioned a number of FP options. I'd like to better understand from your point of view the types of people who choose these various options. For example, how would you describe someone that chooses: [Tailor according to methods identified during listing activity. Probe for FP method 'personification' – e.g., 'appropriate' age, parity, ambitions, fertility desires for each method]
    a. An implant? [Specify duration]

b. An IUD? [Specify copper/hormonal and/or duration, if applicable]
c. The daily pill?
d. Injectables? [Specify Depo IM and DMPA-SC, if applicable]
e. Emergency contraception?
f. Condoms?

Activity 3_ User Characteristics // Interviewer: If users struggle to describe personified methods, use character descriptions, and allow users to match family planning choices to different characters. Give users time to match their FP choices for the different characters/scenarios and probe briefly on various characteristics including SES, marital status, literacy, household composition, employment, lifestyle, religion, rural/urban and how that relates to the choice of family planning method.

Thank you! That was fun getting a better sense of who you think certain methods best suit. Now let's talk about your own experience using FP.

Activity 4_ Journey Maps // Interviewer: For **Qs 5-8**, use the forms for current injectable users and non-users to visualize their experience with previous, current injectable, and other family planning method users (Mapping the experience under the following categories: motivation, information, access, support, experience, and recommendation).

5. What method are you using now, and why? What was the last method used?
    a. [Probe reasons for selection, reasons for discontinuation, reasons for low trust even if using a particular method, people and/or info that influenced their decision]
        i. Did the Coronavirus (COVID-19) pandemic and the social restrictions affect why you chose your current method? Please explain.
    b. What initial questions did you have?
        i. Was there any specific information you were looking for?
    c. Where did you go to get answers for these questions?
    d. Did you talk to anyone about FP or any questions you have around it? Who? Why them/no one?
    e. Where did you obtain your FP method from? Why did you choose that location?
        i. Did the Coronavirus (COVID-19) pandemic and the social restrictions affect why you chose this location? Please explain.
        ii. [For Implant and IUD users only] Did the Coronavirus (COVID-19) pandemic and the social restrictions affect your desire or ability to have your current method removed? Please explain.

SECTION 4: Partner and Family Dynamics

Thank you! As part of this research, we will also be talking to men to get their perspectives on family planning. Along those same lines, we would love to know if or how your partner and family are involved in any part of your family planning process.

Activity 4b_Scenario Probes // Interviewer: Use Twogere scenarios as needed for **Q6** to probe questions on partner and family dynamics.

6. Is anyone else involved in your family planning process or the methods you choose?
    a. [**If yes,** probe for who, role, level of involvement in decision making, their motivations, expectations and considerations around family planning]
        i. [If not spontaneously mentioned, probe for feelings around privacy, discretion, control]
        ii. What kind of information would they be looking for to be better informed on family planning?
        iii. Where would your partner or family go for such information?
    b. [**If no**, probe for reasons]
        i. [If not spontaneously mentioned - Probe for feelings around privacy, discretion, control]
        ii. [If privacy and discretion are mentioned as significant] How do you get information about family planning discreetly and privately?

SECTION 5: Previous and Current Injectable Product Users

7. [**For previous but not current injectable users**] What made you switch from injectables to your current FP method?
    a. What is it about [your current method] that makes it 'right' for you, right now? [Probe for age, life goals/ambitions, parity, fertility desires]
    b. Would you recommend it to others? Why (not)?
    c. Do you plan to continue using this method? Why (not)?
8. [**For current injectable users**] What made you choose an injectable for your FP method?
    a. Where did you find out about injectables as an FP option?
    b. What is it about the injectable that makes it 'right' for you, right now? [Probe for age, life goals/ambitions, parity, fertility desires]
    c. Do you plan to continue using it? Why (not)?

SECTION 6: 4-month DMPA-SC Product

[Interviewer: tailor discussion to reflect experience with injectables, and specifically DMPA-SC]

9. If I were to tell you that an injectable that prevents pregnancy for 4 months may be possible, how would that make you feel?

Researchers at FHI 360 conducted a clinical trial to see if [local name for DMPA-SC/Sayana Press] lasts longer than the label states, and prevent pregnancy for as long as 4 months, rather than 3. Early findings lead us to believe that the same drug and dose used for [local name for DMPA-SC/Sayana Press] is actually safe and effective for 4 months, even though it is only labeled for 3-month use. It is not however approved for 4 months at this time and would require a "new" 4-month product be made available.

We are here today to find out what you think about a potential new 4-month injectable method being introduced in [Uganda/Nigeria]. We would like to know whether or not you would want such a new method and why. We would like to know what kinds of questions you would have around this new injectable method, who you would like to receive that information from, and how such an injectable method could best stand out for you among the other FP methods available to you today.

Activity 5_Potential 4-month User Journey Map // Interviewer: Use form for **potential 4-month product** user journey maps with users to visualize experience

10. Knowing this information, how do you feel about a new 4-month injectable in [Uganda/Nigeria?]? [Probe for positive vs. negative feelings, and reasons why]
    a. Would you consider using this new method? Why (not)?
11. What additional information would you want before using a new 4-month injectable?
12. Where would you like to receive this information? Please mention the 3 most preferred sources.
    a. [Probe: Who would you like this information to come from? (ex. Manufacturer? Ministry of Health (in Nigeria; federal or state-level)? Midwives? Nurses? (In Uganda) District health educator? From other group(s)- specify groups)]?
13. What role would cost play in your decision to use this new injectable? [Probe for acceptability criteria]
    a. [Probe for product price (if applicable/not provided free of charge) and/or service charge(s)].
14. How do you think your peers would react to a new, 4-month injectable? Why is that? [Probe for perceived benefits, drawbacks, and reasons why.]
    a. [If not spontaneously mentioned] What if that meant a user went for reinjection 3 times a year versus 4? [Probe for perceived value re: fewer visits, impact on privacy, opportunity & travel costs]
15. If this new 4-month injectable were introduced in [Uganda/Nigeria], what kinds of questions and/or concerns would you have about it? Why is that?
    a. What kind of concerns do you think your peers might have about this product?
    b. What kind of information would best address these concerns?
    c. Where would you/your peers normally get this kind of FP information?
    d. How should information about this new injectable be communicated to potential users in order to best address these questions/concerns?
16. Would you be more likely to accept an injectable that lasts 3 months or 4? Why is that?
    a. If one of these injectables was proven to have fewer side effects, how would that impact choice? [If appropriate based on users' responses, probe for specifics on type and level of reduction needed for consideration, i.e., the 'tipping point']
    b. If one of these injectables was proven to allow pregnancy more quickly after the method expired in the body, how would that impact choice? [If appropriate based on

users' responses, probe for specifics on how much more quickly the return to fertility needs to be for consideration, i.e., the 'tipping point']

17. Who would be most interested in using a 4-month injectable? Why? [Probe for identification of target user characteristics, drawing from the related discussion of user characteristics above as needed to foster a rich discussion]

SECTION 7: Self-injection

[Interviewer: Section 7 will be carried out using a group discussion format]

[Local name for DMPA-SC] is different from other injectables because it does not have to be administered by a health worker. Rather, users can also self-inject it.

18. [For **current** Sayana Press/DMPA-**SC** users] How do you currently get your [local names for DMPA-SC] injection? Is there a reason why you get it this way?
    a. [Probe for preferences of administration by clinic-based providers, by CHWs [VHTs/CHEWs] and/or by self-administration and reasons why.]
19. [For **non**-Sayana Press/DMPA-SC users, including those using DMPA-IM] From what you know, how do other [local names for DMPA-SC] users get their injection?
    a. Which approach is used the most? Why do you think that is the case?
        i. [Probe for preferences of administration by clinic-based providers, by CHWs [VHTs/CHEWs] and/or by self-administration and reasons why.]
    b. [If not spontaneously discussed] What do you think about self-injection? Why is that?
        i. [If not spontaneously discussed, probe for user concerns/fears around 'correct' administration without health worker oversight; issues of discretion/privacy)]
    c. For women who self-inject, do you think a longer time between injections may affect how comfortable or confident they are in repeating the procedure after 4 months versus 3? [Probe for longer window between training/demonstration and self-administration]

<u>SECTION 8: Expanding market of injectable contraceptives</u>

Activity 7_ Grace Period & Calendar Images // Interviewer: Use the calendar images for the different durations as well as grace periods in question

20. 3-month injectables such as [local name/term for Depo IM] and [local name for DMPA-SC] are already available in [Uganda/Nigeria]. If a 4-month injectable was available as an additional injectable option, do you think there could be problems/confusion among injectable users when they have more choices in terms of how long each injectable lasts? Why or why not?
    a. If yes, what kinds of problems? What are some ways to address or avoid that?
    b. [If not spontaneously mentioned] Do you think there could be problems with confusion among CHWs or clinic-based providers? Why or why not?
        i. If yes, what kinds of problems? What are some ways to address or avoid that?

   c. If you had to describe this new 4-month injectable to your friend in the hopes of convincing her to try it, what would you say? [If not mentioned spontaneously, underscore vis-à-vis existing FP products]
      i. [Probe for what type of messaging will be needed to increase awareness and interest in further injectables]

21. Is it more important to have a method that lasts a long time but gives you a shorter amount of time or 'grace period' to re-inject, or would you prefer a method that lasts a shorter amount of time but gives you a longer amount of time to re-inject, in other words has a longer 'grace period'? And by 'grace period', we mean the days before and after the injection date when it is safe to re-inject. Why is that?

In addition to the 4-month injectable we have been talking about, there are also some injectables at different stages of development that can prevent pregnancy for up to 6 months. One of these is quite far along in the development process and would resemble the [local name for 3-month Depo (IM)] available here in [Uganda/Nigeria] and likely be only administered by a provider. This new 6-month injectable is expected to be as effective as existing injectables. As with [3-month Depo IM/familiar name], some women using this new method may have irregular periods or their periods may stop – and these side effects may last up to 6 months or more. Also, as with [3-month Depo IM/familiar name], the ability to become pregnant may be delayed after the 6 months are over. However, because the dose is lower over the 6 months, the ability to become pregnant might return more quickly and any side effects may be reduced.

Activity 5_Potential 6-month User Journey Map // Interviewer: Use form for **potential 6-month product** user journey maps with users to visualize experience

22. How would you feel about a 6-month injectable being introduced in [Uganda/Nigeria]? Why is that?
   a. What could be some of the positives of this 6-month injectable? What could be the negatives?
   b. Do you think women currently using a 3-month injectable would switch to a 6-month injectable if it were available? If yes, why? How common would this be?
   c. How do you think your peers in general would receive a 6-month injectable in [Uganda/Nigeria]? Why is that?

23. Because an injectable cannot be 'removed', like an implant or IUD [use familiar terminology], some people worry that those that experience bad side effects will have them for a longer time, since the injectable lasts 6 months. Do you feel that this would be a big problem? Why do you feel that way?
   a. What about if a potential user changes their mind and wants to get pregnant before the six months is over? Do you feel this would be a big problem? Why/not?

24. As I mentioned, the 6-month injectable furthest along in development may not offer the potential for self-injection, but other, future injectables may. How important is it to you that new injectables give you the option to self-inject?
25. If you had to describe this new 6-month injectable to your friend in the hopes of convincing her to try it, what would you say? [If not mentioned spontaneously, underscore vis-à-vis existing FP products]
    a. [Probe for what type of messaging will be needed to increase awareness and interest in further injectables]

[Interviewer: Qs 26-28 will be carried out using a group discussion format]

26. If you could only get one new injectable product in the market, would you prefer a 4- or 6-month injectable? Why is that?
    [If not spontaneously mentioned, probe for whether ability to self-inject with the 4-month injectable versus the provider-only administration of the 6-month injectable impacts preference.]
27. From your perspective, what is the ideal/'right' length that an injectable should protect you from pregnancy? Why?
    a. Would the same go for your peers? Why (not)?
28. As an FP user, injectable or not, how many different injectable choices is the 'right' number to have? Why?
    a. How many is too many? Why do you feel that way?
    b. How do we make sure we don't confuse FP users if there are multiple injectables available that last different amounts of time?

Closing

29. In closing, do you have any questions or suggestions for things we should be thinking about when it comes to potentially introducing these new injectables in [Uganda/Nigeria]? Especially one that lasts 4 months.

Thank you very much for your time and the thoughtful feedback you have shared with me today. I understand that this has been a long discussion and it's possible that you might have more thoughts about this as you think more about it later too. We'd like to give you the opportunity to share any further thoughts with us via WhatsApp for a short period of time after the focus group discussion. We may also contact you with additional thoughts or questions from our side as well. Please let me know if it is OK to recontact you. Thank you.

2021

01						
S	M	T	W	[illegible]	F	S
1	2	3	4	5	6	7
8	9	[illegible]	11	12	13	14
15	16	17	[illegible]	19	20	21
22	23	24	25	26	27	28
29	30	31				

02						
S	M	T	W	T	F	S
1	2	3	4	[illegible]	6	7
8	9	10	11	12	13	14
15	16	17	[illegible]	19	20	21
22	23	24	25	26	27	28
29	30	31				

03						
S	M	T	W	T	F	S
1	2	3	4	[illegible]	6	7
8	9	10	11	12	13	14
15	16	17	18	19	20	21
22	23	24	[illegible]	26	27	28
29	30	31				

04						
S	M	T	W	T	F	S
1	2	3	4	5	6	7
8	9	10	11	12	13	14
15	16	17	18	19	20	21
22	23	24	25	26	27	28
29	30	31				

05						
S	M	T	W	T	F	S
1	2	3	4	5	6	7
8	9	10	11	12	13	14
15	16	17	18	19	20	21
22	23	24	25	26	27	28
29	30	31				

06						
S	M	T	W	T	F	S
1	2	3	4	5	6	7
8	9	10	11	12	13	14
15	16	17	18	19	20	21
22	23	24	25	26	27	28
29	30	31				

07						
S	M	T	W	T	F	S
1	2	3	4	5	6	7
8	9	10	11	12	13	14
15	16	17	18	19	20	21
22	23	24	25	26	27	28
29	30	31				

08						
S	M	T	W	T	F	S
1	2	3	4	5	6	7
8	9	10	11	12	13	14
15	16	17	18	19	20	21
22	23	24	25	26	27	28
29	30	31				

09						
S	M	T	W	T	F	S
1	2	3	4	5	6	7
8	9	10	11	12	13	14
15	16	17	18	19	20	21
22	23	24	25	26	27	28
29	30	31				

10						
S	M	T	W	T	F	S
1	[illegible]	[illegible]	[illegible]	[illegible]	[illegible]	[illegible]
8	[illegible]	[illegible]	[illegible]	[illegible]	[illegible]	[illegible]
15	[illegible]	[illegible]	[illegible]	[illegible]	[illegible]	[illegible]
22	[illegible]	[illegible]	[illegible]	[illegible]	[illegible]	[illegible]
29	[illegible]	[illegible]	[illegible]	[illegible]	[illegible]	[illegible]

11						
S	M	T	W	T	F	S
[illegible]	[illegible]	[illegible]	[illegible]	[illegible]	[illegible]	6
[illegible]	[illegible]	[illegible]	[illegible]	[illegible]	[illegible]	14
[illegible]	[illegible]	[illegible]	[illegible]	[illegible]	[illegible]	21
[illegible]	[illegible]	[illegible]	[illegible]	[illegible]	[illegible]	28
[illegible]	[illegible]	[illegible]	[illegible]	[illegible]	[illegible]	

12						
S	M	T	W	T	F	S
			1	2	3	4
5	6	7	8	9	10	11
12	13	14	15	16	17	18
19	20	21	22	23	24	25
26	27	28	29	30	31	

CURRENT GUIDANCE

Three-Month Duration +

One WEEK ‘Grace Period”

2021

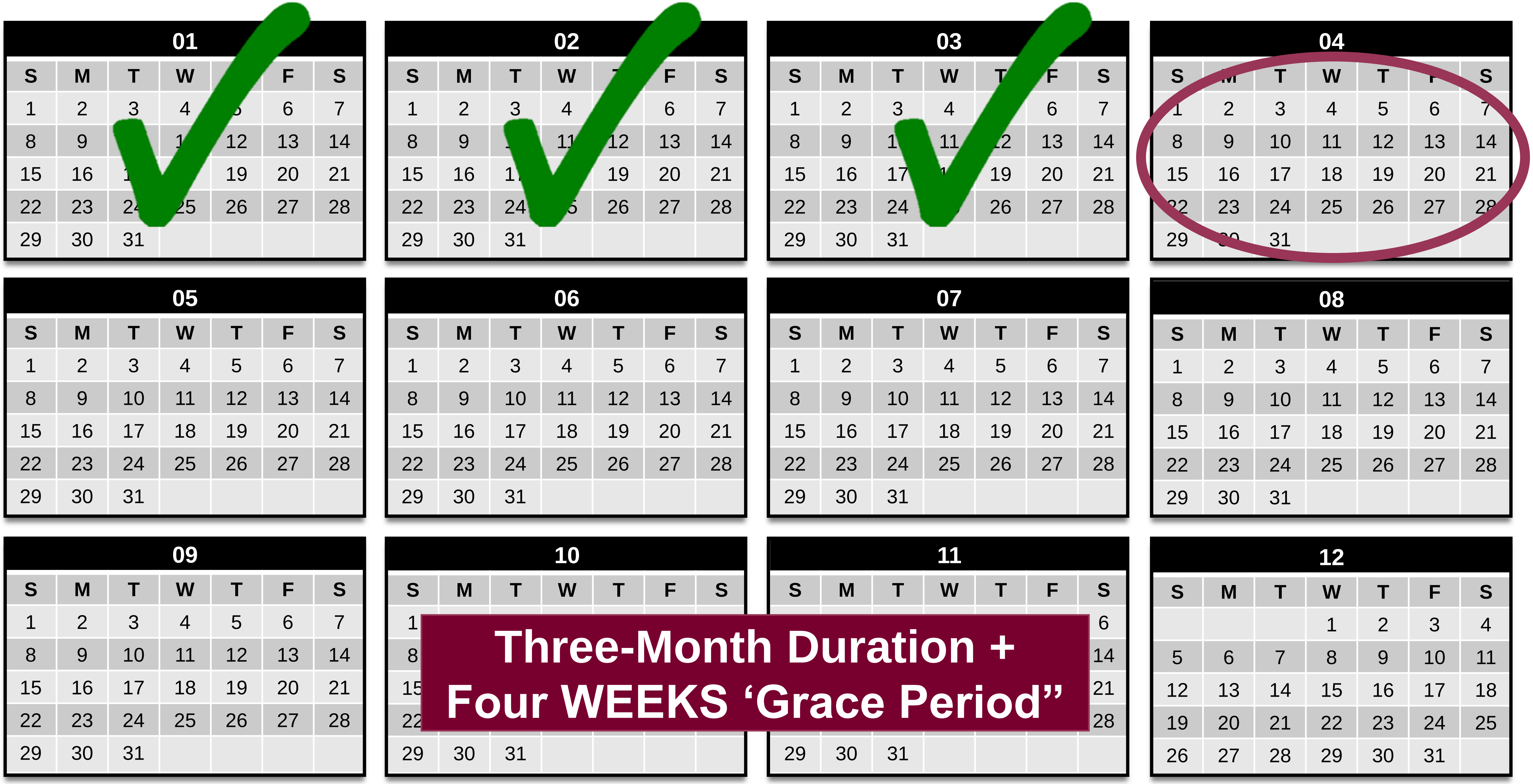

2021

01

S	M	T	W	[illegible]	F	S
1	2	3	4	5	6	7
8	9	[illegible]	[illegible]	12	13	14
15	16	[illegible]	[illegible]	19	20	21
22	23	24	25	26	27	28
29	30	31				

02

S	M	T	W	T	F	S
1	2	3	4	[illegible]	6	7
8	9	[illegible]	11	12	13	14
15	16	17	[illegible]	19	20	21
22	23	24	[illegible]	26	27	28
29	30	31				

03

S	M	T	W	T	F	S
1	2	3	4	[illegible]	6	7
8	9	[illegible]	11	12	13	14
15	16	17	[illegible]	19	20	21
22	23	24	[illegible]	26	27	28
29	30	31				

04

S	M	T	W	T	F	S
1	2	3	4	[illegible]	6	7
8	9	10	11	[illegible]	13	14
15	16	17	[illegible]	19	20	21
22	23	24	[illegible]	26	27	28
29	30	31				

05

S	M	T	W	T	F	S
1	2	3	4	5	6	7
8	9	10	11	12	13	14
15	16	17	18	19	20	21
22	23	24	25	26	27	28
29	30	31				

06

S	M	T	W	T	F	S
1	2	3	4	5	6	7
8	9	10	11	12	13	14
15	16	17	18	19	20	21
22	23	24	25	26	27	28
29	30	31				

07

S	M	T	W	T	F	S
1	2	3	4	5	6	7
8	9	10	11	12	13	14
15	16	17	18	19	20	21
22	23	24	25	26	27	28
29	30	31				

08

S	M	T	W	T	F	S
1	2	3	4	5	6	7
8	9	10	11	12	13	14
15	16	17	18	19	20	21
22	23	24	25	26	27	28
29	30	31				

09

S	M	T	W	T	F	S
1	2	3	4	5	6	7
8	9	10	11	12	13	14
15	16	17	18	19	20	21
22	23	24	25	26	27	28
29	30	31				

10

S	M	T	W	T	F	S
1	[illegible]	[illegible]	[illegible]	[illegible]	[illegible]	[illegible]
8	[illegible]	[illegible]	[illegible]	[illegible]	[illegible]	[illegible]
15	[illegible]	[illegible]	[illegible]	[illegible]	[illegible]	[illegible]
22	[illegible]	[illegible]	[illegible]	[illegible]	[illegible]	[illegible]
29	30	31				

11

S	M	T	W	T	F	S
[illegible]	[illegible]	[illegible]	[illegible]	[illegible]	[illegible]	6
[illegible]	[illegible]	[illegible]	[illegible]	[illegible]	[illegible]	14
[illegible]	[illegible]	[illegible]	[illegible]	[illegible]	[illegible]	21
[illegible]	[illegible]	[illegible]	[illegible]	[illegible]	[illegible]	28
29	30	31				

12

S	M	T	W	T	F	S
			1	2	3	4
5	6	7	8	9	10	11
12	13	14	15	16	17	18
19	20	21	22	23	24	25
26	27	28	29	30	31	

Four-Month Duration +
One WEEK 'Grace Period"
